# Mutuality as a method: advancing a social paradigm for global mental health through mutual learning

**DOI:** 10.1007/s00127-023-02493-1

**Published:** 2023-07-01

**Authors:** Dörte Bemme, Tessa Roberts, Kenneth A. Ae-Ngibise, Nyaradzayi Gumbonzvanda, Kaustubh Joag, Ashraf Kagee, Mercilene Machisa, Claire van der Westhuizen, André van Rensburg, Samantha Willan, Milena Wuerth, May Aoun, Sumeet Jain, Crick Lund, Kaaren Mathias, Ursula Read, Tatiana Taylor Salisbury, Rochelle A. Burgess

**Affiliations:** 1 King’s College London, London, UK; 2 Save the Children, New York, USA; 3 Rozaria Memorial Trust, Murewa, Zimbabwe; 4 University of Edinburgh, Edinburgh, UK; 5 Centre for Mental Health Law & Policy, Pune, India; 6 Stellenbosch University, Stellenbosch, South Africa; 7 University of Cape Town, Cape Town, South Africa; 8 Kintampo Health Research Centre, Kintampo, Ghana; 9 South African Medical Research Council, Cape Town, South Africa; 10 University of Canterbury, Christchurch, New Zealand; 11 University of Warwick, Coventry, UK; 12 University of Kwazulu-Natal, Centre for Rural Health, Durban, South Africa; 13 University College London, London, UK; 14 Burans, Herbertpur Christian Hospital, Atten Bagh, India

**Keywords:** Global mental health, Mutuality, Mutual learning, Decolonizing knowledge, Social determinants of mental health, Epistemic justice

## Abstract

**Purpose:**

Calls for “mutuality” in global mental health (GMH) aim to produce knowledge more equitably across epistemic and power differences. With funding, convening, and publishing power still concentrated in institutions in the global North, efforts to decolonize GMH emphasize the need for mutual learning instead of unidirectional knowledge transfers. This article reflects on mutuality as a concept and practice that engenders sustainable relations, conceptual innovation, and queries how epistemic power can be shared.

**Methods:**

We draw on insights from an online mutual learning process over 8 months between 39 community-based and academic collaborators working in 24 countries. They came together to advance the shift towards a social paradigm in GMH.

**Results:**

Our theorization of mutuality emphasizes that the processes and outcomes of knowledge production are inextricable. Mutual learning required an open-ended, iterative, and slower paced process that prioritized trust and remained responsive to all collaborators’ needs and critiques. This resulted in a social paradigm that calls for GMH to (1) move from a deficit to a strength-based view of community mental health, (2) include local and experiential knowledge in scaling processes, (3) direct funding to community organizations, and (4) challenge concepts, such as trauma and resilience, through the lens of lived experience of communities in the global South.

**Conclusion:**

Under the current institutional arrangements in GMH, mutuality can only be imperfectly achieved. We present key ingredients of our partial success at mutual learning and conclude that challenging existing structural constraints is crucial to prevent a tokenistic use of the concept.

## Introduction

The interdisciplinary field of global mental health (GMH) has promoted evidence-based mental health care in low- and middle-income countries for the past 15 years. Since the inception of the field, the locus of knowledge and power in global mental health has been under scrutiny guided by the question “Whose knowledge counts?” in the production of evidence [1–4]. Despite the goal to develop effective treatments and mental health policies in diverse settings, the field’s priorities, concepts, and outcomes have remained skewed towards the epistemic rules of academia, reflecting its origins as an academic enterprise. While the evidence-based approach has many benefits, the research designs favored by clinicians and epidemiologists may have inadvertently silenced other forms of expertise. Community mental health practitioners, people with lived experience of mental health problems, civil society members, and social scientists are uniquely attuned to the complexities, needs, and challenges in their specific settings. Yet, their expertise is often muted in GMH research design, implementation, and publication practices. Moreover, many indigenous and informal forms of care are not tested to assess their effectiveness and are, therefore, not considered evidence-based. The key question then is: How can diverse actors produce mental health knowledge and care more inclusively and equitably across their epistemic differences? To answer this question, we theorize and operationalize the notion of “mutuality” and how it may engender novel epistemic and collaborative practices in global mental health.

### What is mutuality?

There is no singular definition of mutuality. According to Brown [11], existing definitions share the emphasis on “reciprocal transactions and exchanges, mutual influence and responsiveness, interdependency and a sense of common purpose, exercised in an egalitarian manner” (p. 830). Here, we take a praxiographic [12] that views mutuality as shaped and defined in practice and by multiple knowledge communities.

Calls for greater mutuality in knowledge production have gained traction in global health, emphasizing the need for “mutual learning” [4–6], “mutual capacity building” [7], “shared learning” [8], and “mutual collective accountability” informed by the African philosophy Ubuntu [9, 10]. Here, mutuality is mobilized in efforts to better recognize the expertise and innovation of people living and working in the global South and to decolonize the field’s knowledge practices and partnerships, which have remained underpinned by unequal power dynamics and systematic racism [20–23]. Abimbola and Pai [24] show that academic journals are disproportionately edited by white men in the global North, global health education is concentrated in high-income countries, and prestigious authorship positions, even in publications from international collaborations, are dominated by high-income country researchers. A recent analysis of data movement in global health further suggests a troubling flow of data drawn from people in the global South for analysis by scientists in the North [25]. To the extent that partnerships continue to be necessary, Fekadu and colleagues call for long-term reciprocal relationships grounded in principles of mutual learning, empowerment, autonomy, and freedom, in which all partners are considered active agents for change [6].

Global mental health, more specifically, has long co-evolved with critiques of its evidence base [26–31]. Yet, here too, funding and epistemic power remains largely concentrated in the global North and misaligned with local needs [32]. Leading journals, such as the Lancet Psychiatry, thus now call for mutuality as part of their reflexive turn. In a recent editorial, the editors declare the end and new beginning of GMH by acknowledging editorial mistakes and problematic power dynamics. “Without sufficient forethought”, they write, “global mental health may become a globalised iteration of psychiatry’s potential to reinforce existing power structures and hierarchies” ([5] p. 721). Instead, they call for a new model of knowledge production that recognizes “that expertise comes from individuals and communities in specific social, cultural, and economic environments, rather than the world being a blank slate”, as the rhetoric of the “treatment gap” had suggested [33, 34]. To achieve this change, the editors call for more “mutual learning between communities at a more complex level” [5] (p. 721). Responding to this call, we offer a practice-based theorization of mutuality as a concept and a method of knowledge production. Mutuality, we suggest, has the potential to allow differently situated actors to work more equitably across epistemic differences and power divides. In our case, we brought together 39 collaborators working in 24 countries, whose expertise grounded in qualitative and quantitative research, community mental health practice, and lived experience of mental health problems. Despite our differences, we shared an interest in advancing a “social paradigm” for global mental health, which takes seriously the social, historical, cultural, and contextual factors that shape mental health in place.

We begin by recognizing that mutuality has long been practiced in activist communities, whose knowledge does not necessarily enter the academic knowledge base.^1^ Mutual aid groups, for example, share resources, knowledge, and forge solidarities at the community level, often out of necessity due to adversity and institutional neglect. Activist-scholar Dean Spade defines mutual aid as “a form of political participation in which people take responsibility for caring for one another and changing political conditions […] by actually building new social relations that are more survivable.” ([13], p. 136). During the Covid-19 pandemic, mutual aid became globally more visible and recognized as ‘innovation’, including in community mental health care [14]. Yet, in many parts of the world, it must be recognized as ‘care as usual’ [15–17]. Especially countries that have experienced infectious disease epidemics have a wealth of mutual aid expertise. South Africa, for example, saw the HIV activist movement Treatment Action Campaign (TAC) in the late 1990s and the formation of Community Action Networks (CANs) during Covid-19 [18]. Laying out three principles on how to make mutuality work across age, gender, racialized, and professional groups, the CAN organizers in Cape Town, for example, recommend to: 1) “Focus on critical connections more than critical mass”, i.e., value the quality of relations rather than the number of people connected, 2) “practice collective consciousness,” i.e., decenter the self and focus on collective goals and wellbeing, and 3) “move at the speed of trust,” i.e., recognize trust as the most important “metric that informs action” [19]. These principles hold valuable lessons for any work across epistemic and power differences in global mental health and informed the design of our own mutual learning process.

## Process: how did we design a space for mutual learning?

The “Together to Transform” (T2T) project was initiated by seven researchers at King’s College London (HK, CL, TS, DB, TR, UR) and University College London (RB), who research how mental health is shaped by social forces, including political economy, history, and the specificity of context. This group developed an internal funding bid pursuing the dual goals of transforming GMH methodologically, through mutual learning, and paradigmatically, through promoting the shift towards a social paradigm. When the funding was awarded, these members invited collaborators from their longstanding research partnerships to join. In total, 39 participants working in Afghanistan, Australia, Bangladesh, Canada, Colombia, Ecuador, Ghana, India, Israel, Kenya, Lebanon, Malawi, Mexico, Mozambique, New Zealand, Palestine, Pakistan, Peru, Sierra Leone, South Africa, Spain, Syria, UK, US, and Zimbabwe joined the mutual learning process. As we discuss in greater detail below, by extending the invitation from the global North to the South, and by staying within established academic networks, some key power dynamics that mutuality seeks to challenge were in fact reproduced. Despite these limitations, the aim was to leverage and shift existing institutional power towards practices of power sharing [42] and to create dialogue across three common epistemic divides with unequal power; (a) that between academics from primarily quantitative disciplines (psychiatric epidemiology) and from qualitative or theoretically driven disciplines (sociology, anthropology), (b) that between academics and practitioners working at the community level, and (c) that between collaborators situated in the global North and South.

In the first online meeting, a participatory agenda-setting exercise identified cross-cutting principles (see Table 1) and four themes based on which smaller “pod” groups formed: (1) community mental health systems, (2) scaling up, (3) capacity building and funding, and (4) lived experience, resilience, and trauma. Each participant chose a pod, and all pods included 6–10 people from the global North and South and from multiple professional backgrounds. The goal was to explore the chosen theme and to develop an output together that articulates how global mental health practice needs to change to better address the social dimension of mental health. The pods had an independent budget (750 GBP), defined their own goals and process, and all decisions were made collaboratively. Two co-facilitators from different locations and backgrounds were envisioned to lead each pod, but this model was adapted (see below). Practitioners were compensated for their time in meetings and for preparatory tasks for co-facilitation (25 GBP/hour). Access to university databases, an affiliation with King’s College London and support with internet access were offered. The pods were encouraged to develop a “brave space agreement”, e.g., ground rules to ensure the full and safe participation of all [36]. A facilitation guide designed by a participatory action research methodologist supported the process and the use of creative methods. While these aspects of mutual learning were pre-conceived, the process itself and the meaning of mutuality were shaped in practice over time by all collaborators. For this article, we iteratively distill our arguments from: (1) the written feedback given by all collaborator in an anonymous process evaluation form during our last meeting, (2) written input to drafts of this article from co-authors (who were all participants in this project), (3) pod minutes, and (4) the organizers’ observations across all pod meetings (DB, TR, MW).

**Table 1:** Initial motivation, needs, and priorities of all collaborators, collected through an anonymous intake form and synthesized thematically by DB, TR, and MW after the initial meeting

*Motivations: what motivates you to join the mutual learning process?*
Dialogue between academics, community practitioners, clinicians, and service users
Evidence-based approaches to shift global mental health towards the social
Policy recommendations guiding local level change
A research or position paper
*Needs: what do you need to participate?*
Compensation for time of non-salaried participants and others they want to consult
Compensation for internet connectivity and data where needed
Access to university library sources and databases
Clear and reasonable expectations for time/work involved
Consideration in scheduling for time zones and care responsibilities
*Principles: what should undergird all our processes?*
Co-production with communities and people with lived experience
Intersectoral approach informed by the social determinants of mental health
Commitment to feminist and decolonizing approaches to collaboration
Focus on the community as the origin of mental health and the location of practice/change
Attention to the structural and political factors shaping mental health, policy, and care

## Understanding mutuality from its constraints

It is tempting and customary to showcase successes and then explore limitations. We choose to reverse this order because mutuality is neither easily nor fully achievable once and for all; it is best empirically understood through the structural constraints that curtail its full realization. This perspective brings into view the larger structures that cement hierarchies and the epistemic injustice that mutuality seeks to overcome. We thus start by stating that we have inevitably fallen short of what we set out to achieve.

The first obvious contradiction inherent in this project is that it was initiated by a group of academics from the global North, based in London. This reflects, in part, the funding landscape in which resources are concentrated in well-resourced institutions, with constraints on who can access these resources [37]. Thus, even projects that aspire to more diverse leadership are limited from the outset by inequities in the way that funding structures are designed by and for academics within the centers that already dominate English-speaking research. These UK academics attempted to recognize the limitations of their own perspective through transferring power to the pod groups.

Another clear limitation to mutuality was the selection of participants. Each pod combined the expertise of community mental health practitioners and researchers from diverse global locations. Yet, we recruited participants from existing research networks rather than through an open and transparent process. This was a necessity arising from the short timeframe of the grant. It also recognized the importance of relationships for this type of work, which cannot be established without investment in time and resources [35]. The self-selection process brought together a group of people deeply invested in reflexive, participatory, decolonizing, and co-productive methodologies. This generated a group composition that is not representative of GMH. Several pods held consultations with additional stakeholders drawing on their language skills and relationships to reach out more widely to implementation experts or community mental health workers in rural settings. The independent pod budget allowed them to include voices based on their own priorities.

A consequence of this self-selection process was that it reinforced the hegemony of English, which was without a doubt an exclusionary mechanism. English remained the working language in all pods which was a barrier to full participation due to different levels of proficiency. In addition, differently situated actors use English in different ways. Academics tended to use theoretical language that was not always accessible, which likely had a silencing effect. This was mitigated by the co-facilitators and participants, who actively acknowledged these epistemic differences and made room for everyone. The organizers also created other channels to contribute, such as the Zoom chat function, Padlet exercises, anonymous polls, and invited all to share thoughts via email after meetings.

One key learning was that power sharing is challenging, and that it works best when heavily administratively supported. The goal of power sharing is not simply to decentralize power but to devolve and share it, resisting the urge to take the most expedient path and instead transferring decision-making to the group in an honest attempt at full engagement. Many participants appreciated what they called the “open”, “shared” or “leaderless” facilitation style. Feminist scholar Jo Freeman has argued that “structureless leadership” [40] often masks rather than erases power dynamics. It was thus important to make the informal structures that emerged explicit, and to distribute authority among as many people as reasonably possible, rotate tasks, share information frequently, and invite critique of the process throughout. However, despite their best efforts, the organizers found themselves holding more responsibilities than they had envisioned. Nearly all collaborators operated close to their maximum capacity and were reluctant to commit to the co-facilitator role. The co-facilitation approach was, therefore, adapted according to pod needs. One pod nominated a facilitator with extensive participatory research experience (KM), two opted to rotate the role, and one had no designated facilitator but open group discussions. To create continuity between sessions, the organizers attended all meetings and provided hands-on administrative support, e.g., writing syntheses, scheduling, and briefing the rotating co-facilitators. The feedback shows that this scaffolding administrative support was crucial to the success of the mutual learning process. One member wrote the process might “have fallen apart otherwise. While the absence of designated pod leader was experienced as positive, some also noted the downsides: a lack of direction and difficulties taking collaborative decisions when attendance of pod members was irregular. Some pods took 4 to 5 months to decide on outputs while others changed direction at mid-point. While the slower and non-linear process initially worried the organizers, it turned out to be a key strength of the mutual learning process.

Compensation delays of several months due to non-transparent bureaucratic university processes put a strain on mutuality. The organizers could not reliably predict when the funds would arrive which impacted relationships with practitioners who were distanced from the funding mechanisms and reliant on payments to make ends meet. Mutuality and trust are inextricable from the practicalities of compensation through which the value of someone’s contributions is made indisputable. This is particularly the case for people working outside of research, such as people with lived experience, health practitioners, and freelancers, for whom such activities were not a recognized part of their work but took place in their spare time. The compensation delays reduced motivation for some and diverted research assistance time towards follow-up with finance. Despite these bureaucratic difficulties, the feedback showed that compensation was highly appreciated. One participant wrote: “Compensating everyone’s time also helped. I felt responsible to take time and keep engaging.” Another remarked how compensation is still extremely unusual in “knowledge exchange schemes” and that funders often expect collaborators to give their time “in kind”.

Mutuality also requires us to turn a critical eye on the affordances and material infrastructures of digital teamworking and the unequally distributed challenges. The high cost of connectivity did not only apply to data, phones, and computers. One collaborator wrote: “I live in my village and at times had to drive my 60 km in order to attend an online meeting. Therefore, cost was more than data bundles, but access, time etc.” The hidden burdens of online meetings were also reflected in differential attendance patterns, stability of internet connection, and disruptive environmental factors. Those working directly with communities operated on more socially responsive and unpredictable schedules, while most academic collaborators participated from offices or homes with the resources and predictability this entails. Mutuality was also difficult to achieve and represent online. King’s College London did not allow the listing of collaborator on its web pages unless collaborations were underpinned by formal and financial commitments. This limited the visibility of our collaborations and required us to move out of the university online environment.

While we celebrated our differences and sought to address inequalities, discomfort arose occasionally. Issues were brought up with the organizers who brought them back to the whole group (if necessary, anonymously). Yet, even when issues were resolved by group consensus some voices were de-prioritized. For example, different views prevailed as to whether people with lived experience could be consulted to inform the pod’s work. Some academics felt strongly that formal ethics approval was necessary, while the lived experience advocates and community practitioners in the group felt comfortable reaching out to their established networks. In the end, this pod decided to hold off on consultations since consensus could not be reached. The organizers made additional resources available for a commentary authored by the lived experience member of the pod. This resolution was amicable, but it narrowed the range of voices and sided with the time frame and rules of academic knowledge production, which would have delayed, and potentially limited, broader participation.

## Outcomes: what did we learn from each other? What difference did it make?

Despite the challenges, the mutual learning process succeeded in creating sustainable relations, remarkable shifts in our thinking, and diverse outputs targeting stakeholders in many sites of the GMH assemblage. The pods unsettled and re-imagined dominant scripts and practices in global mental health through a social lens and called to rethink diagnostic categories, funding priorities, scale-up approaches, and to focus on community assets rather than deficits.

More specifically, the pod on *Community Mental Health Systems* reversed the deficit model common in GMH research and articulated a strength-based approach to community mental health care. Drawing on experiential knowledge and primary data from urban and rural settings in Ghana, Palestine, South Africa, and India, members developed comparative case studies of the informal systems of care, such as traditional and faith-based healers, advocacy and peer support, as well as ‘invisible’ practices of care within families that are insufficiently recognized and supported. The pod also took account of the political economy, historical legacies of colonialism, war and conflict, and the demographic profiles of their communities, resulting in an article manuscript [41]. Importantly, practitioners could contribute local knowledge that academics lacked. This changed the tone of group discussions from critiquing current approaches (which ultimately casts doubt on the roles of community-based advocates) to valuing the work that often remains invisible in systems of funding, regulating, and researching mental health care.

The pod on *Scaling* attended to the practices, relations and resources involved in scaling up complex social interventions and conventional mental health care. Scaling is often associated with the replication of evidence-based models across contexts while retaining fidelity. Critics thus view scaling as the expansion of one-size fits all approaches with too little contextual adaptation. This pod develops a resource that made the local knowledge of implementers accessible to further mutual learning. Combining a scoping review with interview-based case studies, they brought together experiential knowledges of community organizations and peer-reviewed evidence. They demonstrated that scaling relies not merely on models but on relationships, trust, an organizations’ values, degrees of flexibility, attention to timeframes of stakeholders and funders, and above all, the meaningful co-production of priorities with communities. They also included implementation failures, which is often overlooked knowledge in GMH.

The pod on *Lived Experience, Resilience and Trauma* explored the limitations and affordances of current concepts of trauma when used as an individualized clinical diagnosis or broader metaphor for collective, intergenerational, and structural suffering in communities experiencing protracted violence and adversity. Conducting a scoping review, the group found that too little is known about the experience of, expression of, and responses to trauma in indigenous and global South communities. Aiming for a collaborative grant proposal, the group highlighted the need to use participatory approaches that center on the lived experience, local idioms, and socio-political ecologies in which trauma unfolds, acquires meaning, and is responded to. This may help to problematize and reframe the distinctly western notions of trauma and resilience used within GMH practice.

The pod on *Capacity Building and Funding* discussed what it takes to better resource community mental health systems. The current funding landscape in GMH evolves around processes and priorities set by funders in the global North, which are insufficiently attuned to local realities at the community level. This pod produced a commentary published in the Lancet Global Health, advocating for more equitable partnerships between funders and community organizations [39].

The mutual learning process also produced cross-cutting outputs, such as two conference panels, one public online event, four audiovisual podcasts, two sets of policy briefs, one lived experience essay, and an interactive website.^2^ The diversity of outputs made us realize the importance of accommodating the needs and incentives of differently situated collaborators and their audiences. Rather than integrating knowledge and producing a singular form of evidence, all pods diversified genres within the limits of their resources.

## Conclusion: key ingredients for mutual learning

Mutuality as a method treats the outcomes and processes of knowledge production as intertwined. Only when all participants can equally contribute, feel heard, make decisions, and determine priorities together can a transformative epistemic space emerge. Mutual learning as a collaborative practice, importantly, does not pursue integration—which can itself be a site of epistemic violence—but generates insights and learnings at the dynamic intersection of different ways of knowing. As such, mutuality cannot be achieved once and for all but is a relational ethos that needs to be co-created and maintained over time. To conclude, we offer the following key ingredients that enabled the relative success of our mutual learning process.

*Involve diverse collaborators and center their needs* As we strove to include as broad a range of stakeholders as possible, it proved pivotal to inquire in detail about everyone’s needs, motivations, and challenges to meaningfully participate in this process (see Table 1). This allowed us to ensure that everyone had sufficient and tailored support (financially, skill-building, digital resources). We also found it helpful to have people in each pod who straddled intersections and could translate between epistemic divides.

*Invest in trust and collective care* While we recognize the exclusionary mechanisms of our group’s self-selection, all pods benefitted from existing relationships, especially because meetings took place online. Many participants noted the surprisingly rapid generation of trust within the pods. Ultimately, the relational strength and trust were the key ingredients to successful mutual learning, echoing the mutual aid principle to “move at the speed of trust” [19]. In the process evaluation, members wrote they appreciated “building relationships that feel like they will last” and “a sense of fellowship.” Another collaborator with a clinical background pointed out that “trust was established in part due to the empathic and sensitive manner of the pod members,” but that rapid trust building also poses risks. They wrote:

“As a consequence of rapid trust building some participants shared fairly personal matters about themselves early on in the process. A concern arose regarding the extent to which participants were making themselves vulnerable by sharing psychologically distressing material with and the responsibility of the pod members to provide a holding space in the event that they became distressed.”

Trust is the key ingredient of mutuality but the corollary of this is that issues of safeguarding and collective care require upfront attention, which in future projects we would place greater emphasis on.

*Allow for slower pace and open-ended, iterative process* Trust building and rotating co-facilitation led to a slower pace and an open-ended, iterative process. All pods placed emphasis on discussing key issues at length, on active listening and longer deliberation before deciding on the direction and outputs to pursue. This was credited with making mutuality work. One collaborator wrote they appreciated “the time to build connections and trust, rather than jumping into output development right away.” Similarly, another participant wrote: “Viewing this as a long-term process made dipping in and out of the meetings feel ok—knowing this was the start of something long-term.” Thus, the meandering and slower pace was an inherent part of building consensus and developing sustainable collaborations [38]. A key learning point was, therefore, to allow a longer timeframe and continuously tweak the process. The participant-driven pace, trust and open-endedness are ultimately what created the desired permeability between disciplinary, epistemic, and power differences. Projects that aspire to mutuality are thus inevitably slower than projects that rely on centralized leadership. Put simply, working across epistemic difference and power imbalances takes time and willingness to listen, as well as to potentially tolerate moments of awkwardness, meandering, and slowdown.

*Focus on unlearning* Importantly, mutual learning requires the unlearning of views and prejudices shaped by coloniality, class, gender, and location. Creating a space for mutual learning is more than promoting the acquisition and production of new knowledge. We invited reflexivity and questioned the systems of oppression that we are part of throughout. Only then could we begin to transform the prejudices that shape current approaches to mental health. The organizers asked for critical feedback throughout, yet the extent to which all participants felt able to raise such challenges was likely variable.

*Measuring mutuality* Mutuality defies universal metrics of success because it is shaped in practice by all who participate in it; as such it specific and situated. In keeping with our epistemic diversity, however, some members suggested measuring mutuality by developing markers against which one could assess whether a project was informed by mutuality. The qualitative colleagues countered this might be “too blunt an approach” that would miss important nuances, while another collaborator highlighted the risks of formalizing it. Mutual learning, they wrote, “is a complex and time-consuming process”, so we should encourage others to “do the best they can to incorporate mutuality rather than not do it because they can’t tick off all the boxes.” All felt strongly that the notion should not be used in tokenistic ways.

To conclude, we propose to understand and operationalize mutuality as a knowledge practice that engages as many epistemic locations as possible and queries where power sits, and how it can best be shared. Under the current institutional arrangements in GMH, mutuality could only be imperfectly achieved. However, as an aspiration mutuality requires us to name and challenge the institutional constraints that curtail its realization to create more inclusive epistemic spaces and processes in the field.

## Data Availability

Not applicable.

## Footnotes

1 Praxiography is a methodology of practice theory driven research. It focuses on the study of practices as the core analytical unit.

2 All outputs can be found on the project website: https://www.together2transform.org/.
